# Interaction of Internal and External Organizations in Encouraging Community Innovation

**DOI:** 10.3389/fpsyg.2022.903650

**Published:** 2022-07-07

**Authors:** Andjar Prasetyo, Bekti Putri Harwijayanti, Muhammad Nur Ikhwan, Moch Lukluil Maknun, Mochammad Fahlevi

**Affiliations:** ^1^Regional Development Planning Agency of Magelang City, Central Java, Indonesia; ^2^Poltekkes Kemenkes Semarang, Central Java, Indonesia; ^3^Regional Development Planning Agency of Pekalongan Regency, Central Java, Indonesia; ^4^National Research and Innovation Agency, Jakarta, Indonesia; ^5^Department of Management, BINUS Online Learning, Bina Nusantara University, West Jakarta, Indonesia

**Keywords:** interaction, internal organization, external organization, community innovation, informal

## Abstract

An area generally has a different character from other regions, including the limited resources that exist in the area. Therefore, it is necessary to strive to encourage human resources through various interactions, and one of the efforts made is the interaction of organizational capacity in the field of innovation. The purpose of this study is to analyze and evaluate the process of organizational interaction in the field of community innovation in Magelang City, Central Java Province, Indonesia. Secondary data sources from the Magelang City Research and Development Agency were 322 innovations from 2004 to 2019, while the primary data came from structured interviews with ten innovators. The findings of the analysis are presented with a qualitative description to evaluate the interaction of innovations in the context of local government organizations. The sampling technique in the study uses accidental sampling. The analysis tool uses participatory exploration that focuses on the government's efforts as an internal organization in encouraging the community as an external organization through the interaction of innovation. The findings of this study are the formation of informal organizations in the community through the interaction of innovations carried out through internal organizational mechanisms. The finding in this study is that the organization's internal interactions in innovation are proven to be beneficial for the community which encourages the growth of community innovation, increases people's income, and contributes to increasing Magelang City's Regional Original Income.

## Introduction

The contribution of innovation to society is growing along with the high global interaction through technology. The convenience of the community in carrying out their activities in the form of economic, social, and cultural activities appears as a result of innovation. Implementation of the contribution can occur due to the organization that manages it. This model of management is shown by the interaction between the government and the community, and with the expected benefit being the community's efforts to solve the problems they face independently, it can be said that buying is better than making. This condition is related to community empowerment, wherein in the process there is an interaction between the subject (government) as the organization and the object (society) as the beneficiary.

The model of community empowerment in Indonesia in its development has been scientifically revealed, for example (Shamadiyah, 2013), community empowerment is directed at developing natural resources and human resources in their environment (Maratade et al., 2016), analyzing the internal and external environment in coastal community empowerment programs (Muljono et al., 2016), and evaluating the performance of twenty locations as an organization, (Fuada et al., 2018), which examined changes in perceptions of the community, or knowledge intervention about iodine can be done by empowering the community. Furthermore (Salatan et al., 2018), with a strategy of empowering fishing communities as an organizational solution, in line with a study also conducted by Yurianto (2019), community empowerment was carried out by establishing new business units, improving the quality of service businesses, capitalization of capital from various sources, and expansion of existing businesses, which is the implementation of the organization in general. Furthermore, the community strategy is a progressive strategy that is strong and has the opportunity to develop an entrepreneurial spirit. There is one most prioritized strategy, which is to encourage the potential of human resources (educated, skilled, enthusiastic, and want to progress) in the agricultural sector as a result of the findings of (Suadnyana et al., 2019). Then (Mar'aini., 2019) explained the Optimization Strategy for the Implementation of the Village Community Empowerment Institutional Program in Community Economic Empowerment. The article from (Hapsari and Mutawali, 2019) also enriches the empowerment literature that raises the study of Chili Agricultural Education Tourism Village Planning with a community-based tourism concept approach. In encouraging poverty alleviation through a community empowerment approach, research conducted by (Sidiq, 2020) states that challenge is the realization of priorities for environmental empowerment and empowering human resources. Furthermore, Tampubolon (2020) is equipped with a study on the empowerment of fishing communities, who have an interest and enthusiasm for entrepreneurship driven by higher needs and cost of living. Interest and enthusiasm for entrepreneurship are shown by community participation in micro, small, and medium business activities. However, the pattern in the research discussed in general has not shown the role or interaction of organizations in a structured sustainable empowerment mechanism.

Meanwhile, the innovation interaction approach can be an alternative scientific solution in Indonesia in the context of community empowerment. Innovation can encourage the human ability to solve problems simply but provide maximum results, and this has also been done in several regions in Indonesia. In Putranto (2016), research has been conducted that discusses the innovation approach through local wisdom in community empowerment. Research (Muhyidin et al., 2017) analyzes the efforts made by the community in implementing development innovation programs and regional empowerment by knowing the form and results of regional development and empowerment innovation programs and (Nurgiarta and Rosdiana, 2019) with studies on community empowerment through the Innovation Program Village. Innovation will be a strategic tool in this important competition for the improvement, creation, and enhancement of businesses to create competitive advantages that are equal to or better than abroad to realize sustainable development (Distanont and Khongmalai, 2020).

The interaction between the government as an organization and the community as the beneficiary, from an organizational perspective, is shown scientifically. For example, in organizations that have worked sustainably, there is an increase in organizational change management on proactive internal change (Lozano and Barreiro-Gen, 2020). However, the complexity of organizational communication in the context of the digital era has resulted in more and more difficult ethical dilemmas that arise in the field of internal and external communication of organizations (Frunza and Grad, 2020). In the leadership aspect, it has also been revealed that effectiveness is needed to revitalize the organization and facilitate adaptation to a changing environment (Karniawati, 2021). A series of innovation policy instruments developed by the government as an organization in the innovation system in Spain and examining the extent to which organizations can support open innovation either by facilitating open innovation practices or by acting on external factors that influence it (Flor et al., 2020) needs to be developed. There needs to be a full participation system of the organization's “internal and external relations” (Shi, 2021). There is a beneficial influence of organizational culture on the innovation development environment and organizational performance in the organizations studied (Schuldt and Gomes, 2020). Structural interaction of external and internal factors within the organization and the classification of external and internal environmental factors in the strategic context of innovation process management also needs to be developed (Shatilo, 2020). Public sector organizations are transforming management styles. Not only is the behavior of government agencies such as businesses, where managers play a central role, but aspects of client service are also becoming more important. The next aspect is communication is the input or message from one person to another. Organizations need communication to streamline their work and carry out tasks in a flawless manner (Kristina., 2020). How organizational functions can lead to integrated responses to efforts to meet community needs and contribute to population well-being (Inoue et al., 2021). Agreement among similar lobbying organizations is a necessary condition for influencing policy-making, while external conflict still provides many opportunities for interest groups to influence policy-making (Truijens and Hanegraaff, 2021). Various types of open innovation manifestations and practice-specific open innovations are applied by organizations (Rumanti et al., 2021). Using internal and external stakeholders contributes to our understanding of radical organizational change as it demonstrates the external impact of change not only on focused sports organizations but also on their external stakeholders, who are critical to organizational survival (Thompson and Parent, 2021).

Some of these studies provide concrete evidence that community empowerment has contributed to improving the quality of society. The approach of innovations managed by the organization both internally and externally has brought improvements from various perspectives. The role of organizations in encouraging society through an innovative approach has a strategic function. However, how the practice can create the capacity and ability of the community to be able to create their own instead of buying has not been discussed through informal interactions. This issue is important to be realized in a scientific approach to the activities carried out by the Government in the context of organizations that organize innovation interactions. This can be clear evidence that the form of government services to the community in a sustainable manner can have benefits. Therefore, this study aims to analyze and evaluate the process of organizational interaction in the field of community innovation in Magelang City, Central Java Province, Indonesia, which is limited to how to carry out internal and external interactions. The expected benefits are providing a scientific basis for community empowerment practices managed by the organization through internal and external interactions of the organization, sharpening literacy about the importance of the informal approach taken by the organization, and enriching the literacy of the organization's efforts in community empowerment in the field of innovation and other relevant fields.

## Method

This research method is descriptive and qualitative. With the research location in Magelang City, the data were obtained in the period from 2004 to 2020. The secondary data source from the Research and Development Agency of Magelang City was 322 innovations from 2004 to 2019, while the primary data came from interviews with ten innovation actors in Magelang City. The respondent's consideration is based on the character of the owner of the work who has the potential to experience difficulties in describing the proposed work. Qualitative arguments are judged to refer to what, how, when, where, and why something becomes the essence and atmosphere. Qualitative research, thus, refers to the meanings, concepts, definitions, characteristics, metaphors, symbols, and descriptions of things (Lune and Berg, 2017). The sampling technique is non-probability, uses accidental sampling, the population has infinite properties, and the sample is made up of ten innovators whose information can be extracted.

The approach taken with participatory research begins with identifying research questions, gathering information to answer questions, analyzing and interpreting information, and finally in the form of sharing results with participants.

This participatory data analysis refers to Lune and Berg (2017) who explain that promoting emancipatory praxis in participating practitioners, that is, promoting critical awareness that manifests itself as well as practical action to promote change. The different aims of this action research approach are, first, an attempt to increase the closeness between the everyday problems faced by practitioners in a particular setting and the theories used to explain and solve problems; in other words, attempts to unify book theory and knowledge with real-world situations, problems, and experiences. The second objective is to assist practitioners in lifting the veil of their hazy understanding and help them to better understand the underlying issue by increasing their collective consciousness.

## Results and Discussion

Magelang City is an area located in Central Java Province, Indonesia. This city is at the crossroads of Semarang City, Salatiga City, Purworejo Regency, Temanggung Regency, and the Province of the Special Region of Yogyakarta. It is located in the Magelang Regency area. Public interest in the field of innovation already has an adequate innovation ecosystem in terms of the organization that handles it and the quantity of innovation produced every year. The interaction of innovation is based on several dynamic regulations that occur in Indonesia. From 2004 to 2018, the regulations used referred to Law Number 18 of 2002 concerning the National System of Science and Technology, which were then changed to Law Number 19 of 2019 National System of Science and Technology and which is still in use until 2020. Law Number 11 of 2019 concerning the National System of Science and Technology has been ratified as the basis for the implementation of Science and Technology in Indonesia where in this regulation both central and regional governments recognize, respect, develop, and preserve the diversity of traditional knowledge, local wisdom, natural resources, biological and non-living nature, as well as culture as part of national identity.

The role and position of Science and Technology have the following objectives:

a. promote and improve the quality of education, research, development, assessment, and application of science and technology that produces inventions, namely the inventor's idea which is poured into a specific problem-solving activity in the field of technology in the form of a product or process, or improvement and development of a product or process and innovation is the result of thinking, research, development, assessment, and/or application, which contains elements of novelty and has been applied and provides benefits, economic and/or social;
b. increase the intensity and quality of interactions, partnerships, and synergies between elements of Science and Technology Stakeholders;
c. increase the use of Science and Technology for sustainable national development, quality of life, and community welfare; and
d. increase the independence, competitiveness of the nation, and the attractiveness of the nation in the context of advancing the nation's civilization through international relations.

To find a solution to problems faced by the region, technical, functional, business, socio-cultural, and aesthetic viewpoints and/or contexts were combined, and added values from products and/or production processes for the welfare of the people were generated. The Central Government and Regional Governments are required to develop Inventions and Innovations. The form of implementation is in the form of an award from the Central Government, Regional Government, and/or the public to the inventor or one or several people who jointly implement the ideas that are poured into the activities that produce the Invention. Every citizen has the same right to participate as an inventor.

The City of Magelang as a City that commits to the development of Inventions and Innovations is constantly making changes by adjusting the dynamics of the evolving regulations. Since 2004, the City of Magelang has carried out invention and innovation activities. From an organizational perspective, from 2004 to 2008, the organization that handled the echelon III structure with the title Research Development and Report Control Division was the Magelang City Planning Agency, and then from 2008 to 2016, the organization that handled it developed into the Research Development and Development Office, Magelang City Statistics although still with echelon III structure. From 2017 to 2021, due to the arrangement of the Organizational Structure and Work Procedure of the Regional Government, the institution in charge was transformed into the Research and Development Agency for the City of Magelang. Since 2017, it turns out that the only Regional Government at the City Regency level in Central Java Province wasin Magelang City, which has a Research and Development Agency organization with an echelon II structure.

If you look closely, this correlates with the general picture of Magelang City. Magelang City is part of Central Java Province with an area of 18.54 km^2^, which is the smallest area in Central Java Province. In terms of administrative area, Magelang City is divided into three sub-districts. North Magelang District, Central Magelang District, and South Magelang District, with a total output of seventeen urban villages. In 2021, the number of residents sourced from (Datago, 2021Datago., 2021) reached 122,375 people with a male population of 60,221 people and a female population of 62,154 people. A total of 86,372 people belong to the age group of 15 to 64 years or can be said to be in the productive group. The number is divided into 42,645 males and 43,727 females (Datago, 2021Datago., 2021).

Furthermore, the innovation interaction mechanism has been carried out by the Magelang City Government since 2004. Community Invention and Innovation is an activity of providing incentives to the wider community who have inventor ideas, which are poured into a specific problem-solving activity in the field of technology in the form of products or processes, or improvements and product or process development, and which has the result of thinking, research, development, assessment, and/or application, which contains elements of novelty and has been applied and provides benefits, economic and/or social.

This activity is a mechanism for advancing and improving the quality of Education, Research, Development, Assessment, and Application of Science and Technology, increasing the intensity and quality of interactions, partnerships, and synergies between elements of Science and Technology Stakeholders, increasing the use of Science and Technology for the development of national sustainability, quality of life, and community welfare; and increasing the independence, competitiveness of the nation, and the attractiveness of the nation in the context of advancing the nation's civilization through international relations.

In its development, it has gone through various changes, both in terms of the organization that handles it and the stages carried out. However, in general, the mechanism for this innovation process includes implementation from socialization to follow-up after the implementation of innovation interactions. The targets to be achieved in Community Invention and Innovation activities are to increase awareness and raise public awareness in producing and utilizing science and technology to support their activities so that competitive products can be produced.

**Figure 1:**
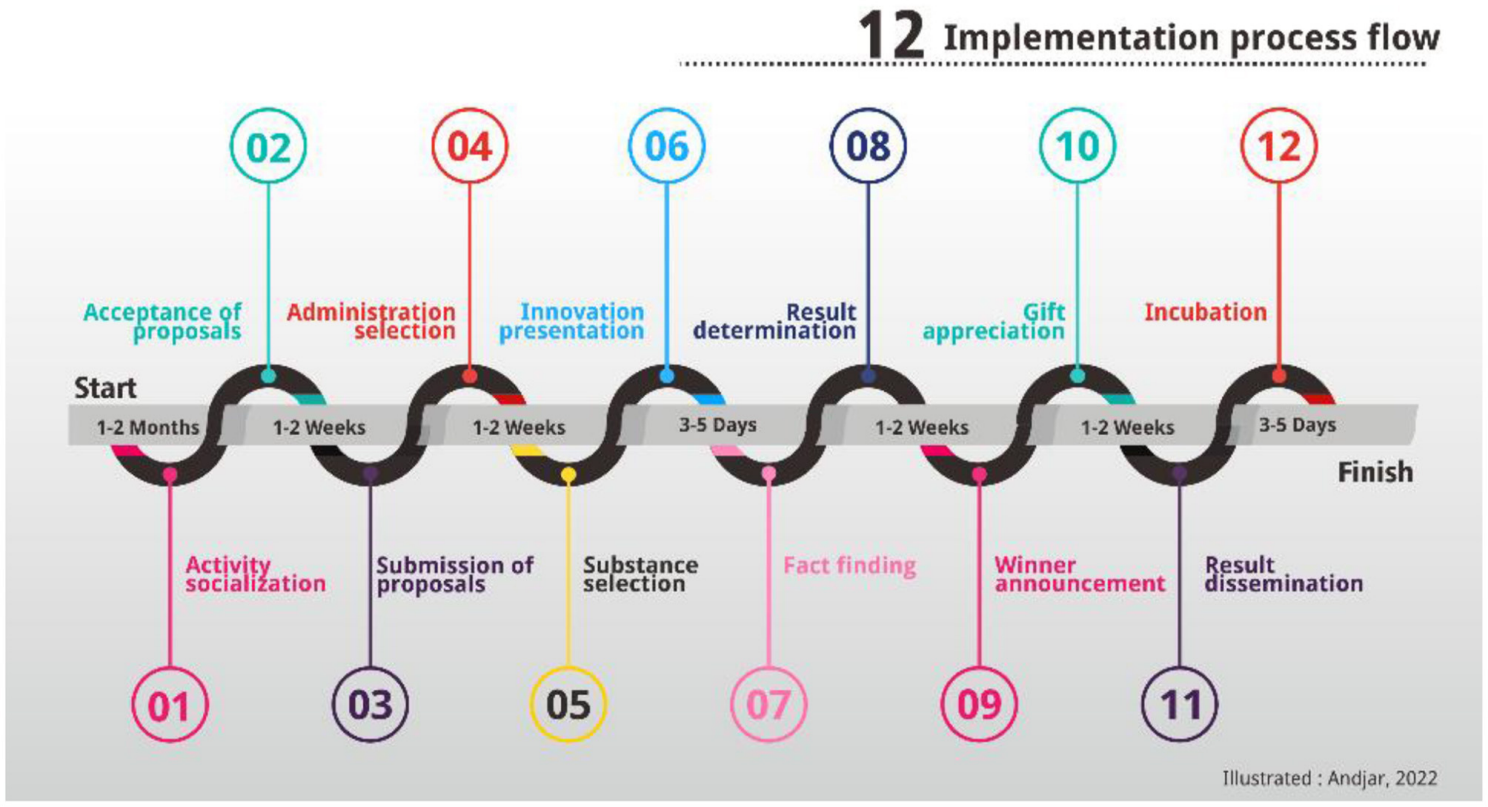
Community innovation interaction mechanism.

Figure 1 provides clarity on the mechanism of innovation interaction carried out by the Magelang City Government toward the Magelang City community.

Figure 1 shows the 12 stages carried out in the context of innovation interaction between the Magelang City Government as an internal organization and the community as an external organization. The community empowerment process occurs in the early stages (first stage-third stage) and the final stage (11th stage and 12th stage). Efforts to empower the community in the early stages were carried out through socialization involving various instruments, both formally and informally. In this stage, Magelang City is facilitated because of the character of the administrative area which consists of three sub-districts, thus facilitating the socialization process. The mechanism that has been carried out since 2004 with the institutional form and the amount of the budget disbursed by the Magelang City Government has resulted in a response from the community, as can be seen in Table 1.

**Table 1:** Identification of innovation interaction results.

**No**.	**Year**	**Form of organization**	**Budget (Rp. Million)**	**Community response**
1.	2004	Field	38.5	9 works
2.	2005	Field	35	1 works
3.	2006	Field	41,445	9 works
4.	2007	Field	43	13 works
5.	2008	Office	45	19 works
6.	2009	Office	72	12 works
7.	2010	Office	72.5	18 works
8.	2011	Office	63.48	29 works
9.	2012	Office	65	24 works
10.	2013	Office	44	35 works
11.	2014	Office	50	17 works
12.	2015	Office	73.5	15 works
13.	2016	Office	78	19 works
14.	2017	Agency	88	39 works
15.	2018	Agency	93	32 works
16.	2019	Agency	134	31 works

*Source: Rnd Agency of Magelang City, 2021*.

From Table 1, it can be seen that there are fluctuations in the form of organization that started from the Division then developed into an Office, and finally formed the Agency, but due to the limited financial capacity of the region in terms of the budget, fluctuations occurred from year to year. Furthermore, the community response provides results that prove the existence of community empowerment with innovation interactions, even though the quantity fluctuates every year. The work in the form of innovation shown is also in line with the concept of innovation, for example referring to Hwang and Choi (2017), which explains the influence of culture which is one of the supporters of innovation and people's behavior. Innovation as understood together is a process carried out by a person or a group of people to produce a product or service to provide convenience in solving a problem that has been encountered by the people of Magelang City. Innovation can contribute to development, which is corroborated by the statement of Pansera and Martinez (2017), who developed a critical analysis of innovation discourse, arguing that a more contextual understanding of the challenges of innovation for development.

Field of invention and innovation community who discover, create, and apply a work of science and technology in the fields of Food and Agribusiness; Health and Medicine; Energy; Transportation; Environment; Fisheries and Maritime Affairs; Information Technology; Education; Crafts and Home Industry, and Social. The general criteria for participating in Community Inventions and Innovations are Indonesian citizens, internal residents proven by ID cards or domiciled in the area of activity, and regional government employees, individually or in groups; and not directly related or affiliated with the organization organizing the activities. The proposed criteria refers to the results of the Community, both individuals/groups, which are poured into a specific problem-solving activity in the field of technology in the form of a product or process or the improvement and development of a product or process; the result of thinking, research, development, assessment, and/or application, which contains elements of novelty and has been applied and provides benefits, economic and/or social; have never received an award either at the Regional or Central level, unless there has been a significant improvement/improvement; easy to disseminate, adopted by the community can be applied at the household scale; the raw materials used are locally based, environmentally friendly; and the scale of investment and management is affordable to the community and has sustainable benefits.

Aspects of Community Invention and Innovation based on the development of science and technology have indicators of increasing the development of science and technology, increasing mastery of science and technology, and having an impact on the development of science and technology for the region. Economic Aspects with indicators consist of an increase in inventors' income, improving the welfare of local communities, and increasing regional economic growth. Social Aspects with indicators include the suitability of community capacity with technology, suitability of local culture with technology, and changes in people's positive attitudes.

The mechanism implemented by the organization uses a community empowerment strategy, with innovation interactions because it is carried out in total with openness, and collaborating with relevant stakeholders shows that there are at least four core elements of holistic innovation, “strategic”, “total”, " open”, and “collaborative,” interrelated with organic integration (Chen et al., 2018). Innovation affects increasing regional advantages that the City of Magelang has adapted due to its limited natural resources, and this is in line with the article from Distanont and Khongmalai (2020) that innovation will become a strategic tool in important competition for improvement, creation, and improvement of business, to create competitive advantage the same as or better than those abroad in the context of realizing sustainable development. Previously even (Do (2016) proved that the empirical findings obtained revealed that the two determinants of technological output, knowledge, innovation, and creative output have a positive effect on competitiveness.

After the Magelang City Government organization conducted innovation interactions in 2004, the form of community empowerment that emerged was the number of people who responded to the innovation interaction, which is presented in Table 2.

**Table 2:** Community responses to the interaction of innovation in Magelang City in 2004–2019.

**No**.	**Year**	**Community response**	**Gender**	
			**Male**	**Female**
1.	2004	9 works	7 people	2 people
2.	2005	1 works	1 person	–
3.	2006	9 works	5 people	4 people
4.	2007	13 works	15 people	5 people
5.	2008	19 works	15 people	5 people
6.	2009	12 works	11 people	4 people
7.	2010	18 works	19 people	2 people
8.	2011	29 works	33 people	12 people
9.	2012	24 works	24 people	12 people
10.	2013	35 works	21 people	62 people
11.	2014	17 works	10 people	9 people
12.	2015	15 works	9 people	8 people
13.	2016	19 works	16 people	7 people
14.	2017	39 works	43 people	7 people
15.	2018	32 works	42 people	6 people
16.	2019	31 works	28 people	7 people

*Source: Research and Development Agency for the City of Magelang, 2021*.

In Figure 1, the innovation interaction mechanism has been explained, where the community empowerment process occurs in the early and late stages. Community empowerment efforts are carried out at an early stage through an informal approach, which is then able to increase the role of the community. In various articles, it has been concluded that community empowerment is an effort that directs the community to be better in a sustainable manner, and the article from being empowered has also been applied in the organizational scope in the form of companies that can improve performance, as mentioned in (Yin et al., 2019), and how to practice empowerment affects firm performance by describing the moderating role of empowerment practices in the relationship between employee–employer exchange characteristics and firm performance. This concept is in line with the informal efforts made by an organization in the Magelang City Government; the community's response at first was still difficult to adapt, but after an informal approach from year to year always appeared works from the community both in quantity and quality.

Over time, in 2017, the Ministry of Home Affairs developed an incentive scheme with a regional innovation index called the Innovative Government Award as part of the elaboration of Government Regulation Number 38 of 2017 concerning regional innovation. This scheme has similarities with the interaction mechanism carried out by the Magelang City Government because the work (Table 1) produced after participating in the selection is given an award in the form of money and a regional head certificate, followed by participation in national activities. The impact of this innovation interaction mechanism then developed with the addition of the Regional Incentive Fund (DID), which was received by the Magelang City Government in 2020 because it was named the Most Innovative City. This DID can be interpreted as a form of local revenue source that can be used for development in Magelang City. This condition is in line with an article that discusses the performance of sustainable innovation on the economy of an inner region (Rauter et al., 2018).

The organization's informal approach to innovation interaction consists of several stages, such as:

a. Organizational interaction in the form of socialization is carried out at the beginning of the activity by providing an innovative interaction mechanism. The people involved in this socialization are representatives of the kelurahan apparatus and several residents in the said kelurahan area. The result is that this innovation interaction is distributed to the community as evidenced by the response to participation. At the socialization stage according to Figure 1, then an assessment of the works that have met the requirements is also carried out. These conditions are completed at the time the assistance is carried out.
b. Interaction mentoring for people who have work, in this case, consists of two models, passive assistance, namely the community goes to the companion, and active assistance, namely the assistant who visits the community who has the work. The mentoring process is carried out by discussing the work owned, followed by documenting the work owned. The output of this assistance is a document containing a description of the work owned by the community. In documenting the work, which also adjusts the community who owns the work, if it cannot be written, the assistant will help compile a description of the community's work. The result is a Community Creativity and innovation Bulletin that has existed from 2004 to 2019.
c. Interaction tracing potential intellectual property (IP): As a form of facilitation of the work owned, a search is also carried out whether the work has the potential for IP registration. This registration is a form of protecting the IP of the public. The result is the protection of IP for several works owned by the public.
d. Interaction dissemination of works. The interaction of innovation at this stage is to provide information related to the work owned by the public. The model used is in the form of a collection of community works and holding meetings with relevant stakeholders by informing the character of the work they have, such as the purpose, benefits, specifications, advantages, economic value, and development prospects. The result is a response from the business world to participate in commercializing the work produced.
e. Interaction production of prototypes. Five works every year since 2017 are facilitated to realize community works. This is done, generally, the work that is owned still has some weaknesses from a scientific point of view, so it needs to be perfected as a work that scientifically has fulfilled its rules. The scientific approach is carried out by involving universities that have the capacity and ability to work that is realized in the form of a prototype. The result is a prototype belonging to the community that is stored in the innovation gallery (see Table 3).
f. Interaction exhibition of innovation products. Pushing for more use of the interaction of innovations carried out by participating in innovation exhibitions at the City, Provincial, and National levels. In addition, it is also exhibited in the gallery which is provided in collaboration between the government and the community. The result is an increase in the capacity of works that are increasingly recognized and utilized.
g. Interaction of Provincial level competition test: After going through a series of screenings conducted by the Magelang City Government, several works were selected to be included in the innovation competition at the provincial level. The submitted works are by the categories given by the Province, not on the results of the assessment that has been carried out by the Magelang City Government. The result is in the form of incentives and awards for the owner of the work from the Provincial Government. In Table 4, it can be seen from the information on the acquisition of awards starting from 2010, although since 2004 they have received awards from the Province of Central Java.
h. Interaction capacity building: After having a prototype with scientific refinements, it is then directed to become a startup by accessing schemes facilitated by Ministries and Institutions. This capacity increase is carried out starting from the formation of startups to the commercialization process. The result is the establishment of a startup company that produces works to be commercialized.
i. Interaction with vendors is done by bringing together companies that have an interest in the commercialization of works owned by the community. The result is an increase in the production of works produced by the community according to the needs and demands of the company.

**Table 3:** Works upgraded to prototype.

**No**.	**Year**	**Nama of work**
1.	2018	Smart On-Grid Actuator
2.	2018	Restrainer Baszain
3.	2018	Gray Water Park
4.	2018	Gepoktan
5.	2018	Umbaran Lipat
6.	2019	Rusend Cut
7.	2019	Mobile Netdesk
8.	2019	Automatic Drying Room
9.	2019	Outdoor Medical Examination
10.	2019	Ketel Pemanas

*Source: Research and Development Agency for the City of Magelang, 2021*.

**Table 4:** Works that won the provincial innovation award.

**No**.	**Tahun**	**Name of work**
1.	2010	Kartu Karakter Perpustakaan
2.	2011	Sludge Substitusi Paving
3.	2012	Jemuran Otomatis
4.	2013	Kain Tenun Serat Pisang
5.	2014	Tabela Cerdas
6.	2015	Kain Krenova Warna Alam
7.	2016	Matcha Cinta Matematika
8.	2017	Umbaran Lipat
9.	2018	Bantur Grill

*Source: Research and Development Agency for the City of Magelang, 2021*.

The stages of an informal approach that runs gradually and sustainably has an impact on increasing community sensitivity in innovation interactions. The results of the approach in the interaction of informal interviews with people who have innovations can be described as follows:

a. Newsqita is a fertilizer product derived from the fermentation of fruit and vegetable waste and then processed into organic fertilizer. In the informal approach process, initially, resistance emerged from the owner of the work due to a lack of confidence in the process of interaction of innovations carried out, but the process was still carried out and the result was that the product was able to provide added value for the owner of the work.
b. Smart On-Grid Actuator (SOGA) has the function of utilizing sunlight with dynamic technology following the direction of the sun from sunrise to sunset, and the harvest of direct sunlight can be consumed by its users. In the interaction, the innovations carried out cannot express the concept in a narrative that is required for every participant who submits his work. After taking an informal approach, the preparation process is facilitated until it becomes a proposal that can demonstrate technical and economic advantages. SOGA is a process of harvesting solar energy into electricity with an automatic system that dynamically follows the direction of sunlight from morning to evening which is directly connected to the installed electrical installation. The SOGA prototype is currently installed at Balitbang Magelang City and can function properly with electricity usage efficiency reaching 10–30%.
c. Farmers Group Generator, namely Gepoktan: Gepoktan is a community innovation that has the main function of producing energy by utilizing water, which is converted into electrical energy by storing batteries. This tool can be used in agricultural areas as a source of renewable energy.
d. Restrainer Bazain, a tool that has the main function as a means to facilitate the slaughter of cows or the like can also be used as a means of artificial insemination, and cow Health care, without stressing the cows.
e. Gray Water Park: The increase in population has the consequence of increasing the burden of pollution in water bodies. The habit of people who dispose of kitchen, washing, and bathroom waste directly into water bodies without going through processing is one of the causes of high levels of pollution in water bodies. On the other hand, the decreasing potential of water resources in Magelang City makes the water scarce at certain times. Therefore, an alternative water innovation is needed to meet the need for water that does not need to be potable (drinking). Treatment and utilization of washing, kitchen, and bathroom waste (graywater) is one of the innovations to meet water needs other than the need for bathing, washing, and drinking (potable). The use of graywater is highly possible to be applied by the community because the amount of graywater production is quite large (50–70% of the total wastewater production) and the pollutant concentration is not too high compared to waste mixed with WC wastewater.
f. Umbaran Fold: The community is increasingly active in channeling ideas that can be used to solve the problems they face. The concept of the idea that is raised is not in the form of a complicated and complex form of innovation, but is simple but able to solve the problems at hand. This innovation interaction can empower the community, and this is shown by the emergence of startups and the existence of collaboration with vendors who have a concern for the work produced.

## Conclusion

Through interaction, innovation is proven to be a model of community empowerment. The interaction of innovation is carried out through several stages starting with socialization, assistance to the community, tracing the potential for IPR protection, dissemination of the work that has been produced even though it requires refinement, prototype production facilitated by the government, product innovation exhibitions at the City, provincial and national levels, competency test in provincial level competition, and capacity building carried out at the startup level and commercialization with vendors. Another result found is that the interaction of innovation in an informal approach proves that it can benefit the community, encourage the growth of community innovation, increase people's income, and contribute to increasing Magelang City's Regional Original Income.

The ability of the community to dedicate their innovations generally cannot build a description of the ideas they have, but with the organization's internal interactions with people who have innovations, they can provide benefits that can be followed up on social, economic, and cultural terms. The community becomes able to express innovation through informal internal organizational interactions. This condition shows that the role of internal organizations with an informal approach can provide benefits to the community as an external community.

## Data Availability Statement

The original contributions presented in the study are included in the article/supplementary materials, further inquiries can be directed to the corresponding author/s.

## Author Contributions

All authors listed have made a substantial, direct, and intellectual contribution to the work and approved it for publication.

## Conflict of Interest

The authors declare that the research was conducted in the absence of any commercial or financial relationships that could be construed as a potential conflict of interest.

## Publisher's Note

All claims expressed in this article are solely those of the authors and do not necessarily represent those of their affiliated organizations, or those of the publisher, the editors and the reviewers. Any product that may be evaluated in this article, or claim that may be made by its manufacturer, is not guaranteed or endorsed by the publisher.
